# A community proposal to integrate proteomics activities in ELIXIR

**DOI:** 10.12688/f1000research.11751.1

**Published:** 2017-06-13

**Authors:** Juan Antonio Vizcaíno, Mathias Walzer, Rafael C. Jiménez, Wout Bittremieux, David Bouyssié, Christine Carapito, Fernando Corrales, Myriam Ferro, Albert J.R. Heck, Peter Horvatovich, Martin Hubalek, Lydie Lane, Kris Laukens, Fredrik Levander, Frederique Lisacek, Petr Novak, Magnus Palmblad, Damiano Piovesan, Alfred Pühler, Veit Schwämmle, Dirk Valkenborg, Merlijn van Rijswijk, Jiri Vondrasek, Martin Eisenacher, Lennart Martens, Oliver Kohlbacher

**Affiliations:** 1 European Molecular Biology Laboratory, European Bioinformatics Institute (EMBL-EBI), Cambridge, CB10 1SD, UK; 2 ELIXIR Hub, Cambridge, CB10 1SD, UK; 3 Department of Mathematics and Computer Science, University of Antwerp, Antwerp, 2020, Belgium; 4 French Proteomics Infrastructure ProFI, Grenoble, (EDyP U1038, CEA/Inserm/ Grenoble Alpes University) Toulouse (IPBS, Université de Toulouse, CNRS, UPS), Strasbourg (LSMBO, IPHC UMR7178, CNRS-Université de Strasbourg), France; 5 ProteoRed, Proteomics Unit, Centro Nacional de Biotecnología (CSIC), Madrid, 28049, Spain; 6 Biomolecular Mass Spectrometry and Proteomics, Bijvoet Centre for Biomolecular Research and Utrecht Institute for Pharmaceutical Sciences, University of Utrecht, Utrecht, 3548 CH, Netherlands; 7 Netherlands Proteomics Center, Utretcht, 3584 CH, Netherlands; 8 Analytical Biochemistry, Department of Pharmacy, University of Groningen, Groningen, 9713 AV, Netherlands; 9 Institute of Organic Chemistry and Biochemistry, Czech Academy of Sciences, Prague 1, 117 20, Czech Republic; 10 CALIPHO Group, SIB Swiss Institute of Bioinformatics, Geneva, 1015, Switzerland; 11 Department of Human Protein Science, Faculty of Medicine, University of Geneva, Geneva, 1205, Switzerland; 12 National Bioinformatics Infrastructure Sweden (NBIS), SciLifeLab, Department of Immunotechnology, Lund University, Lund, 223 62, Sweden; 13 Proteome Informatics Group, SIB Swiss Institute of Bioinformatics, Geneva, 1015, Switzerland; 14 Computer Science Department, University of Geneva, Geneva, 1205, Switzerland; 15 Institute of Microbiology, Czech Academy of Sciences, Prague 1, 117 20, Czech Republic; 16 Center for Proteomics and Metabolomics, Leiden University Medical Center, Leiden, 2333 ZA, Netherlands; 17 Department of Biomedical Sciences, University of Padova, Padova, I-35121, Italy; 18 Center for Biotechnology, Bielefeld University, Bielefeld, 33615, Germany; 19 Department of Biochemistry and Molecular Biology, University of Southern Denmark, Odense M, 5230, Denmark; 20 Interuniversity Institute for Biostatistics and Statistical Bioinformatics, Hasselt University, Hasselt, 3500, Belgium; 21 Center for Proteomics, University of Antwerp, Antwerpen, 2000, Belgium; 22 Applied Bio & Molecular Systems, VITO, Mol, BE-2400, Belgium; 23 Netherlands Metabolomics Centre, Utrecht, 3511 GC, Netherlands; 24 Dutch Techcentre for Life Sciences / ELIXIR-NL, Utrecht, 3511 GC, Netherlands; 25 Medical Bioinformatics, Medizinisches Proteom-Center, Ruhr-University Bochum, Bochum, 44801, Germany; 26 VIB-UGent Center for Medical Biotechnology, Ghent, 9052, Belgium; 27 Department of Biochemistry, Ghent University, Ghent, 9000, Belgium; 28 Applied Bioinformatics, Department of Computer Science, University of Tübingen, Tübingen, 72074, Germany; 29 Center for Bioinformatics Tübingen, University of Tübingen, Tübingen, 72074, Germany; 30 Quantitative Biology Center, University of Tübingen, Tübingen, 72074, Germany; 31 Biomolecular Interactions, Max Planck Institute for Developmental Biology, Tübingen, 72076, Germany

**Keywords:** proteomics, mass spectrometry, computational proteomics, databases, bioinformatics infrastructure, data standards, training, multi-omics approaches.

## Abstract

Computational approaches have been major drivers behind the progress of proteomics in recent years. The aim of this white paper is to provide a framework for integrating computational proteomics into ELIXIR in the near future, and thus to broaden the portfolio of omics technologies supported by this European distributed infrastructure. This white paper is the direct result of a strategy meeting on ‘The Future of Proteomics in ELIXIR’ that took place in March 2017 in Tübingen (Germany), and involved representatives of eleven ELIXIR nodes.

These discussions led to a list of priority areas in computational proteomics that would complement existing activities and close gaps in the portfolio of tools and services offered by ELIXIR so far. We provide some suggestions on how these activities could be integrated into ELIXIR’s existing platforms, and how it could lead to a new ELIXIR use case in proteomics. We also highlight connections to the related field of metabolomics, where similar activities are ongoing. This white paper could thus serve as a starting point for the integration of computational proteomics into ELIXIR. Over the next few months we will be working closely with all stakeholders involved, and in particular with other representatives of the proteomics community, to further refine this paper.

## Introduction

Proteomics is generally defined as the large-scale experimental study of the proteome. High-throughput proteomics approaches have matured significantly, becoming an increasingly used tool in biological research. The rapid development of the field over the last decade has been primarily driven by technological progress in mass spectrometry instrumentation, chromatographic separation, genomics (increased availability of sequenced genomes) and bioinformatics ^1, 2^. The primary workhorse of proteomics today is mass spectrometry coupled to liquid chromatography (LC-MS), with less commonly used high-throughput proteomics approaches based on antibodies (e.g., protein arrays and other immunofluorescence-based techniques). Key applications of proteomics are the study of (differential) protein expression in time and space, characterization of protein primary structures and their post-translational modifications (PTMs), such as phosphorylation and glycosylation, elucidating protein structures, and protein-protein interactions. It is the primary technology driving progress in unravelling signalling networks (e.g. protein phosphorylation driven signalling) and protein interaction networks, and is indispensable for understanding biological function of protein isoforms and disentangling their specific functions. In complex systems biology and systems medicine studies, proteomics often complements information gained from other omics levels, such as genomics and transcriptomics (the so-called proteogenomics and proteotranscriptomics studies ^3^), metagenomics (metaproteomics), glycomics, and metabolomics.

As already highlighted, remarkable advances in computational methods have been a key driver in the fast development of the field of proteomics. ELIXIR ( https://www.elixir-europe.org/) is a European Research Infrastructure (ESFRI), which coordinates, integrates and sustains bioinformatics resources across its member states. Some of the most prominent research groups in proteomics are active in Europe, such as Prof. Matthias Mann (Martinsried, Munich, Germany), Prof. Ruedi Aebersold (Zurich, Switzerland), Prof. Albert Heck (Utrecht, the Netherlands) and Prof. Mathias Uhlén (Stockholm, Sweden). In addition, Europe also hosts worldwide renowned groups that are focused on the development and application of widely-used bioinformatics tools and resources, including MaxQuant ^4^, the OpenMS framework ^5^, CompOmics ^6^ tools, such as PeptideShaker ^7^, the PRIDE database, as the world-leading proteomics repository ^8^ (also coordinating the global ProteomeXchange Consortium of proteomics resources ^9^), and a collection of open data standards and related software ^10^, emphasizing the European leading role in the activities of the HUPO (Human Proteome Organisation) Proteomics Standards Initiative (PSI).

Outside mass spectrometry proteomics, the Human Protein Atlas ^11^, located in Sweden, is the world-leading resource for antibody-based characterization of the human proteome. In this context, it should also be highlighted that two of the three sites behind the development of UniProt ^12^, the most-widely used protein knowledgebase, are European: the Swiss Institute of Bioinformatics (SIB) and the European Bioinformatics Institute (EMBL-EBI). Furthermore, neXtProt ^13^, which is the reference knowledgebase for human proteins in the context of the HUPO Human Proteome Project, is also developed and maintained at SIB.

Additionally, it is of note to highlight that a number of national proteomics-dedicated infrastructures have already prioritised structuring and developing computational proteomics among their activities. This is the case of the French proteomics infrastructure ProFI (which has, for instance, devoted a major investment to develop the Proline tool) and the Spanish infrastructure ProteoRed (which has, for example, contributed heavily to PSI activities). Also, proteomics is represented in other national scientific infrastructures. One example is the Netherlands DTL (Dutch Techcentre for Life Sciences), having an active role in advocating for FAIR data management.

**Figure 1.**
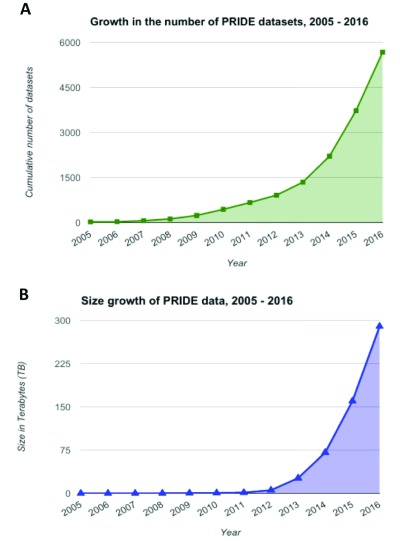
Number of datasets ( **A**), and the total size of the PRIDE database ( **B**), from 2005 to 2016. Data was retrieved directly from the PRIDE Archive Oracle ^TM^ database instance, which contains the file sizes and the dates when the datasets where originally submitted.

In the context of providing infrastructure for storing proteomics data, it is worth highlighting here that, although it was not the case just a few years ago, thanks to many of these efforts, and with the support of scientific publishers and funders, public availability of proteomics data has increased exponentially in recent years, becoming a common scientific practise, similarly to how it routinely happens in disciplines such as genomics and transcriptomics ^14^. Figure 1 summarises the growth of the PRIDE database in recent years.

Although the European bioinformatics community has been very active in the proteomics field (see above), proteomics activities have not been highly represented in ELIXIR so far. There was a proteomics component in two small ELIXIR pilot actions (collaborations between EMBL-EBI, now ELIXIR central node, and Bioinformatics Services to Swedish Life Science [BILS], now ELIXIR-Sweden). In addition, a selection of proteomics tools and training events have been included in the ELIXIR tool registry ^15^ ( http://bio.tools) and in TeSS, the ELIXIR training portal ( https://tess.elixir-europe.org/), respectively. These platforms were recently presented to the proteomics community ^16^. However, we propose that, due to the growing importance of the field and the prominence of proteomics bioinformatics activities in Europe, it is the right time to formally integrate proteomics activities in ELIXIR.

In this context, in February 2017, EMBL-EBI and ELIXIR-DE initiated the first ELIXIR ‘Implementation study’ involving proteomics approaches, as a starting point for the field. Within this implementation study, suggested by ELIXIR management, the meeting “The future of proteomics in ELIXIR” took place in Tübingen (Germany), as a general strategy meeting for future proteomics activities in ELIXIR. In this white paper, we first summarize the main conclusions of the meeting, and then explain possible future directions for the incorporation of proteomics activities in ELIXIR, taking into account the current overall ELIXIR structure, split in platforms and use cases.

## Methods

### Meeting “The future of proteomics in ELIXIR”

The meeting took place on March 1 ^st^–2 ^nd^ 2017 in Tübingen (Germany). Attendance was widely advertised through ELIXIR dissemination channels (e.g., mailing lists, newsletter) and was open to any interested member of the community. There were 24 attendees representing eleven ELIXIR nodes: Germany (host), Belgium, Czech Republic, Denmark, France, Italy, Netherlands, Sweden, Switzerland, EMBL-EBI, and one representative from the ELIXIR Hub. The detailed minutes of the meeting are available as Supplementary File 1. The meeting started with a presentation given by Rafael Jiménez (ELIXIR Chief Technical Officer) who provided a general overview of the current ELIXIR activities. This initial talk was followed by a series of presentations where the representatives of each node summarized their ongoing activities related to proteomics. All the presentations are freely available at http://tinyurl.com/elixir-proteomics.

The remainder of the meeting was devoted to an open discussion on how to bring together activities, experience, stakeholders, and emerging needs. First of all, ten potential ELIXIR stakeholders in this domain were identified, namely: funding agencies, regulatory bodies, educators, infrastructures, publishers, core facilities, bioinformaticians, life scientists, industries and hospitals/patients. Second, a series of needs and challenges were outlined for each of the stakeholders, and these were then mapped to each of the existing ELIXIR platforms: *Data*, *Tools*, *Interoperability*, *Compute* and *Training* (see below). The output of this activity is summarized in Supplementary Table 1. In the second day of the meeting, more concrete topics, derived from the identified needs and challenges were outlined by the attendees, and then organised in wider areas, so called “clusters”. Finally, they were prioritised, with the idea that these could form the basis for future proteomics activities in ELIXIR.

### Current ELIXIR internal structure

**Figure 2.**
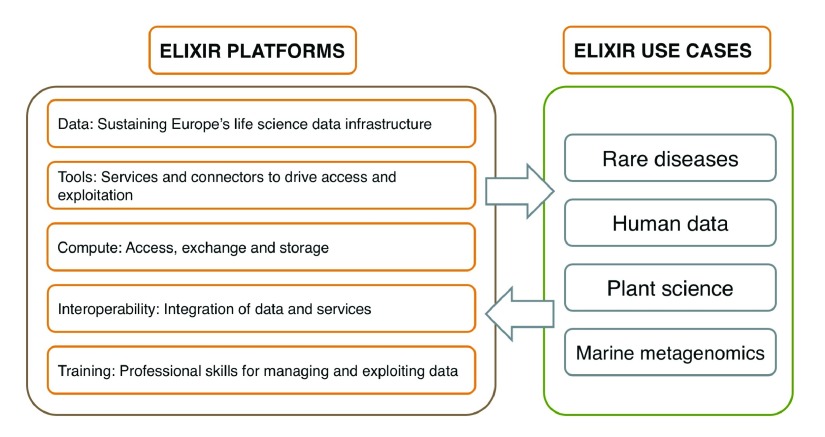
Overview of current ELIXIR platforms and use cases (by May 2017).

Here we describe the current status of ELIXIR platforms and use cases, by May 2017. ELIXIR’s activities are structured around platforms and use cases. They bring together resources and expertise from the ELIXIR Nodes and form the basic unit of operation within ELIXIR. The ELIXIR platforms are responsible of the implementation of the ELIXIR programme and are organised in five key areas: *Data*, *Tools*, *Compute*, *Interoperability* and *Training*. The platforms are complemented by four use cases that represent four scientific communities: *Human data*, *Rare diseases*, *Marine metagenomics* and *Plant sciences* ( Figure 2). The use cases drive the work of the ELIXIR platforms by defining their bioinformatics needs and requirements. The close collaboration between the ELIXIR use cases and platforms ensures that the services developed by the ELIXIR platforms are fit for purpose. Each platform and use case is led by a group of senior scientists from across the ELIXIR nodes. In addition to the funding available in each national ELIXIR node, the main source of financial support for ELIXIR activities comes from the ELIXIR-EXCELERATE EU H2020 project. Additional activities are funded through other complementary grants as well as ‘Implementation studies’ supported by the ELIXIR Hub.

***The ELIXIR platforms.*** The *Data* platform focuses on sustaining Europe’s life science data infrastructure. This platform is working on guidelines and indicators for data resources to improve their impact and sustainability ^17^. It also works on improving links between curated data resources and literature data.

The *Tools* platform is dedicated to services and connectors to drive access and exploitation of bioinformatics research software. The main key activities within this platform are centred to facilitate the discovery, benchmarking and interoperability of software. It does also focus on software development best practices, as well as on a strategy for workflows and software containers.

The *Interoperability* platform supports the discovery, integration and analysis of biological data. Activities driven by this platform are organised in projects around identifiers, metadata standards and linked data. It also works on the description of interoperability services as well as specialised workshops named BYOD (Bring Your Own Data) ^18^ to improve the “FAIRness” (Findable, Accessible, Interoperable and Re-usable) of data resources ^19^.

The *Compute* platform is dedicated to the compute, storage, transfer, authentication and authorization of biological data relying on services provided by ELIXIR nodes and e-infrastructures. Finally, the *Training* platform aims to increase the professional skills for managing and exploiting data. Part of activities are meant to train researchers, trainers and service providers, but it also includes other activities related to e-learning, to improve the discovery and availability of training materials and to measure the impact of training.

***Current ELIXIR use cases.*** There are four use cases. First of all, the *Human data* use case develops long-term strategies for managing and accessing sensitive human data. The *Rare disease*s use case supports the development of new therapies for rare diseases. The *Marine metagenomics* use case works towards a sustainable metagenomics infrastructure to nurture research and innovation in the marine domain. Finally, the *Plant sciences* use case develops an infrastructure to support genotype-phenotype analysis for crop and tree species.

## Results and Discussion

### Areas prioritised in the meeting

In an attempt to assess the relative priorities of the various areas of proteomics that might steer integration into ELIXIR, attendees voted on the relative importance of the topics. The following list shows the top-ranked areas for future ELIXIR related proteomics activities (called “Clusters” from now on), sorted in descending order by the number of votes received:

***Cluster 1. Multi-omics approaches.*** It includes topics such as data integration of proteomics and other types of omics data, correlation between gene and protein expression, and development of data standards for “multi-omics” data types. A closely related group of activities was “Cancer proteomics”, comprising topics such as support for clinical proteomics data (including large patient cohorts) and cancer “multi-omics” (proteogenomics) studies.

***Cluster 2. Proteoforms and PTMs.*** The term proteoform ^20^ represents the different molecular forms in which the protein product of a single gene can be found, including changes due to genetic variations, alternatively spliced RNA transcripts and PTMs, among other events. This cluster of activities included topics such as the handling, validation of proteoforms and creation of standards for their description, improvement of the existing connection between proteoforms, genes and metabolites (topic related to Cluster 1 above), and activities devoted to explain unidentified spectral signals.

***Cluster 3. Quality Control (QC) activities.*** In any analytical discipline, QC is essential. Due to the fact that proteomics is a newer and still rapidly developing field, QC has historically not been as well-developed in proteomics as in, for instance, the more established small molecule mass spectrometry field ^14^. This cluster is therefore focused on activities to develop automatic and reliable pipelines for QC of proteomics data at different levels.

***Cluster 4. Data analysis workflows and cloud computing.*** The concrete activities outlined here could be summarized as the development of robust, reproducible, scalable, user-friendly, integrated, QC-controlled, data analysis pipelines, ideally enabling the use of compatible cloud infrastructures, which in addition, could also be used for data storage. Infrastructure supporting efficient development of such pipelines and workflows is also important, including tool repositories and documentation, workflow management systems and interfaces for accessing computational resources.

***Cluster 5. Protein quantification and statistics.*** This topic encompasses the improvement of protein inference in shotgun proteomics approaches, the use of peptides that match to more than one protein precursor, and enhanced data integration/harmonisation for quantitative proteomics. It was perceived by many attendees that, although many of these issues are often considered solved, there are still many improvements possible in this area.

***Cluster 6. Metadata, standardisation, annotation, and data management.*** This topic also includes the improvement in the annotation of proteomics datasets, in particular in data repositories like PRIDE (to facilitate public data reuse by third parties), the development and/or extension of existing Laboratory Information Management Systems (LIMS), standard data formats, and guidelines summarising best practises for data management, following FAIR principles.

The rest of the areas discussed got only one or two votes from the attendees, and included activities related to interactomics, structural proteomics, metabolomics, metaproteomics, the development of benchmarking datasets, and training efforts. Finally, it is worth highlighting an additional proposal for the creation of a repository for tool-related ideas.

### Alignment between ELIXIR activities and the needs of the proteomics community

As mentioned in the ‘Methods’ section, ELIXIR activities are currently structured in five platforms ( *Data*, *Tools*, *Interoperability*, *Compute* and *Training*) and four longitudinal use cases ( *Human data, Rare diseases, Marine metagenomics* and *Plant sciences*) ( Figure 2). Our preferred option is that proteomics becomes the main focus of one additional ELIXIR use case in the near future. If there is no scope for proteomics to have its own use case, other options could be possible, for instance the integration of proteomics activities into the existing ELIXIR use cases. We think that the current use cases, heavily focused on genomics data, would also benefit from having a “multi-omics” perspective. Out of the existing cases, *Human data* would be an obvious choice. The other three could also benefit from proteomics activities: *Rare diseases* (clinical proteomics), *Marine metagenomics* (metaproteomics), and *Plant sciences* (plant proteomics).

In any case, and without considering specific use cases, it is clear that there are several topics that, in our opinion, would fit very well into the scope of the current five ELIXIR platforms:

1- *Data* platform. Metadata, standardisation, annotation and data management activities (Cluster 6), and “multi-omics” approaches (Cluster 1), involving data integration efforts from different omics data types, would be highly relevant in this context. Moreover, QC efforts (Cluster 3) are essential for all such types of data re-use. Indeed, data re-use is a very important aspect in proteomics data, as only 30–40% of the acquired data is typically exploited ^14^. This creates extremely exciting opportunities for proteomics data re-use with specialized tools that can lead to the discovery of new biological information ^21^.

2- *Tools* platform. In this platform, the overall aim would be to increase the visibility, quality and sustainability of proteomics software developed following best practices. First of all, more proteomics tools should be effectively included into the ELIXIR tool registry, and highlighted there appropriately. However, it is worth highlighting that there are around 350 tools proteomics tools represented in this resource already. In addition, the development of improved and user-friendly quantification algorithms and tools, along with direct coupling to dedicated and performant statistical analysis (Cluster 5), also connects directly to the ELIXIR tools platform. Other possible activities would be related to Cluster 4, and would involve the improvement of the description and sharing of proteomics workflows, facilitating the encapsulation of workflows, data and tools into proteomics software containers that could be shared across ELIXIR, taking advantage of existing resources. Finally, the proposed idea in the meeting to create of a repository for tool-related ideas, would also be applicable in the wider ELIXIR context.

3- *Interoperability* platform. Obviously, the activities of this platform are very relevant in the case of “multi-omics” approaches (Cluster 1), for instance the development of data standards for these type of approaches, e.g. to enable better data integration and visualisation. A close connection can also be made to metabolomics with regards to QC (Cluster 3), as the main technology of choice (mass spectrometry) is shared between these two fields.

4- *Compute* platform. Workflow analysis pipelines and activities related to the development of cloud infrastructures (Cluster 4) and QC activities (Cluster 3) should be highlighted here. This would involve different pieces of infrastructure supporting the efficient development of such workflows, e.g. the EDAM ontology and the ELIXIR tool registry ^15^, open data formats, and workflow management systems, among others.

It is important to note that, although originally set up in the *Data* platform, the proteomics ELIXIR implementation study mentioned in the ‘Introduction’ section aims to make a first step forward in this direction, for the popular shotgun (MS/MS) approaches. As a proof of concept, these pipelines will be deployed first in the EMBL-EBI “Embassy Cloud”, a cloud infrastructure based in the Open Stack operating system, with the idea that in the future they can be made available in other cloud systems (e.g. Amazon EC2, Google Cloud, Microsoft Azure), so that the developed pipelines can be freely reused by any interested researcher in the community. In the context of the implementation study, the pipelines are connected to the PRIDE database, bringing the analysis tools closer to the data as datasets become larger in size and complexity. This trend is ongoing for other omics technologies in the context of ELIXIR: for instance, the pipelines developed in the context of the *Marine metagenomics* use case ^22^.

5- *Training* platform. ELIXIR is already working actively in coordinating training activities across Europe (e.g. the already mentioned TeSS portal). Although training was not specifically highlighted as one of the main areas for future development during the meeting, the reason is that everyone assumes that this is implicitly a key need in all bioinformatics fields. While excellent training courses and workshops have already been created in the field, a higher degree of coordination across Europe should be achieved for bioinformatics training activities, and in particular for computational proteomics topics, possibly in coordination with other fields such as metabolomics (see next section).

### Connection to metabolomics and other ESFRIs

As mentioned already, metabolomics and proteomics share a common experimental platform: mass spectrometry. A parallel effort is currently ongoing to integrate metabolomics activities in ELIXIR. There are many topics of common interest where both fields could benefit from a closer interaction: e.g. the development of common software (for QC, data visualisation, signal processing, to name but a few) and open data standards. Some initiatives are already working towards this goal, e.g. the computational mass spectrometry group ( http://compms.org/), and some recent activities recently carried out by the PSI, but a lot of work remains to be done. One concrete proposal would be to coordinate efforts concerning training activities. ELIXIR would be an ideal platform to enable this coordination effort.

Coming back to the proteomics field, PRIME-XS ( http://www.primexs.eu) was a four-year Infrastructure project funded by the EU FP7 Programme (2011–2015), coordinated by Prof. Albert Heck. The main aim of PRIME-XS was to provide funded access to an infrastructure of state-of-the-art proteomics technology to the European biological and biomedical research community. It is anticipated that future tailored EU H2020 infrastructure grant calls might provide an opportunity to create a second iteration of this successful project. The potential synergies with ELIXIR, considering the outlined activities in this white paper, would be obvious at different levels, for instance the provision of a cloud computing infrastructure to store and analyse the acquired data in a scalable and reproducible way, and the development of pipelines to allow data integration between proteomics and other omics data types.

## Conclusions

We hope that this white paper acts as a guide to achieve the overall goal of integrating proteomics into ELIXIR. In our opinion, this would not only make perfect sense from a scientific point of view (proteomics information provides an essential ingredient in a “full picture” of life), but also because it would enable computational proteomics to work in close contact with bioinformatics activities in other high-throughput fields, which will undoubtedly trigger many possible interactions, exchanges, and exciting novel developments.

## Supplementary material

Supplementary File 1: Detailed minutes of the meeting *“The future of proteomics in ELIXIR”.*

Supplementary File 2: Table S1: Table summarising the output of the open discussion at the meeting.
