# Supplementary material for: A community proposal to integrate proteomics activities in ELIXIR
Source: F1000Res. 2017 Jun 13;6:ELIXIR-875. [Version 1] doi: 10.12688/f1000research.11751.1 (PMC5499783; doi:10.12688/f1000research.11751.1)
Supplement: Supplementary file 1 [file f1000research-6-12693-s0000.tgz › 896688d9-3de3-4617-a794-573b03fb7cbd.pdf]

“The future of proteomics in ELIXIR”

Minutes of the meeting (Tuebingen, Germany, March 1-2, 2017)

- Agenda
  - Day 1 - March 1
  - Day 2 - March 2
    - Discussions
- Minutes
  - Day 1
    - Elixir node requirements, ideas and suggestions
      - Session 1
        - Notes/Questions
      - Session 2
        - Notes/Questions
    - Interactive session
      - Identification of Stakeholders
      - Needs and Challenges
        - Group 1: Funding agencies, Regulatory bodies, Educators
      - Group 2: Infrastructures, Publishers, Core Facilities
      - Group 3: Bioinformaticians, Life Scientists
      - Group4: Industry, MS industry, Patient
  - Day 2 Notes
    - Integration within Elixir
    - Recommendations
    - Priorities
      - Paper
    - Discussions
    - Wrap-up
      - The future of proteomics in ELIXIR

Agenda

Day 1 - March 1

Time	Topic	Presenter
12.00-13.00	Lunch buffet	
13.00-13.15	Welcome and introductions	Juan A. Vizcaíno & Oliver Kohlbacher
13.15-13.45	General introduction to ELIXIR and context for proteomics activities	Rafael Jimenez
13.45-15.10	**Elixir node requirements, ideas and suggestions - session 1**	
13.45-14.00	• ELIXIR-DE	Oliver Kohlbacher and Martin Eisenacher
14.00-14.15	• ELIXIR-EBI	Juan A. Vizcaíno
14.15-14.25	• ELIXIR-BE (pdf version)	Lennart Martens
14.25-14.35	• ELIXIR-CH	Frederique Lisacek
14.35-14.45	• ELIXIR-CZ	Martin Hubalek and Petr Novak
14.45-14.55	• ELIXIR-DK	Veit Schwämmle
14.55-15.10	• Questions and Answers	
15.10-15.40	Coffee break	
	**Elixir node requirements, ideas and suggestions - session 2**	
15.40-15.50	• ELIXIR-FR	Myriam Ferro, CEA/Grenoble, France
15.50-16.00	• ELIXIR-IT	Damiano Piovesan, Padua / Padova
16.00-16.10	• ELIXIR-NL	Peter Horvatovich and Merlijn van Rijswijk

16.10-16.20	• ELIXIR-SE	Fredrik Levander
16.30-16.35	• External ideas and suggestions	
16.35-16.50	• Questions and Answers	
16.50-17.00	Common task: White paper	Juan A. Vizcaíno
	**Interactive session**	
17.00-18.30	Needs, challenges and alignment with ELIXIR<br>• Stakeholders – all – 15’<br>• Needs and challenges – 4 groups – 40’ (30’D + 10’R)<br>• Alignment with Platforms and Use cases – all – 15’<br>• Priorities – all - 20’<br>• Proteomics use case – 3 groups – 40’ (30’D + 10’R)	Rafael Jimenez (facilitator), everyone
19.30	Dinner at Ludwigs (Commute/walk)	

Day 2 - March 2

Discussions

09.00-10.00	Needs, challenges and alignment with ELIXIR<br>• Alignment with Platforms	Groups
10.15-10.15	Recommendations (white paper + use case)	All
10.30-10.45	Coffee break	
10.45-11.00	Priorities	
11.00-12.00	Discussion topics<br>• Alignment with other disciplines<br>• Engagement with external stakeholders (non-ELIXIR)<br>• ...	All
12.00-12.30	Wrap up	

Minutes

Day 1

Rafa Jimenez gave a presentation about the general structure of ELIXIR activities, including the general information about the current platforms and use cases, the current funding, and plans for the near future.

Elixir node requirements, ideas and suggestions

The present ELIXIR-node representatives gave flash talk on their node's requirements, ideas and suggestion. The audience was able to gather questions for the following section.

Session 1

Notes/Questions

- ELIXIR-CH
  - Where did the paxDB expression information come from?

A

release	1.0	2.1	3.0	4.0
organisms	10	12	30	53
publications	10	31	55	90
samples	28	99	187	414
spectral counts	16 mio	41 mio	135 mio	2030 mio
proteins covered	70'535	85'452	188'183	> 300'000

B

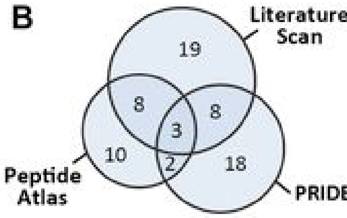

doi/10.1002/pmic.201400441

  - Why NeXprot does not import from PRIDE and just PeptideAtlas?
    - We were asked by HPP to ensure <1% global FDR at protein level and we did not know how to ensure that using PRIDE datasets
    - However, we do provide links to PRIDE
    - In the future it will be possible (for those projects represented with mzIdentML to reproduce the 1% FDR level. Using the PRIDE Cluster approach is difficult to produce a reliable FDR value.
    - Place for an implementation project in the future?
    - Belgium already reprocesses large sections of PRIDE data (e.g., all human data, or all mouse data) with state-of-the-art tools (SearchGUI with three search engines and PeptideShaker) at 1% FDR, and the results

obtained have already been used in several published manuscripts, including annotations of genome and transcriptome databases.

- ELIXIR-DK
  - Are the tools so far presented registered in bio.tools?
    - The tools provided by the de.NBI network are registered in bio.tools with “Collection: de.NBI”, subset of BioInfra.Prot tools: limit to “Credits: BioInfra.Prot”)
    - List of proteins resources could be used in the white paper. Link to bio.tools with all the proteomics resources?
  - If I register a tool in http://ms-utils.org/ does it show automatically in bio.tools?
    - It is a push model
  - Is there any connection with the http://omictools.com website?

- De.NBI/ELIXIR-DE
  - Consulting/analysis as a service highlighted as a priority by some de.NBI-service centers (BioInfra.Prot, CIBI). At the moment ELIXIR does not offer consulting as a service. Could this be interesting to introduce in ELIXIR?

Session 2

Notes/Questions

- ELIXIR-FR
  - ProLine system: really great tool; great opportunity also for creation of online training materials! How modular is it with regards to the processing algorithms used? I.e., can you plug in a new search engine, quant. algorithm, or statistical processing method?
    - Modularity is one of the strengths of the Proline tool. Functionalities were developed in independent Java/Scala libraries. New tools/algorithms can easily be implemented by wrapping existing libraries or by using the Proline database directly (using SQL queries). Another way to interoperate with Proline is through the use of WebServices and JMS based communication. We have already started to integrate the SearchGUI tool in the MS-Angel one, which is a module of the Proline software suite.

            - The system thus creates a great hub for implementation of various tools developed, and making these available to (non-bioinformatics expert) users, especially with the cloud interface!
    - Are you using the CWL for your workflow system (ProteoRE)?
        - No because 1) the CWL is not ready (It has just been upgraded at version 1.0), 2) not usable in Galaxy as it is now (support for the alpha version) See here on GitHub.

- ELIXIR-NL:
    - Great idea to have data stewardship built-in, with the aim to support research projects at the moment of application. This is a model worth copying! Is this support provided to anyone asking for it, or are there criteria for who gets the help?
        - Data Stewardship is (recently) a mandatory component of Grant Applications of our National Science Foundation, which you can also budget the costs for. Many Universities and University Medical Centres are setting up (internal) help desks, for their researchers. DTL is bringing these initiatives together to learn from each others and exchange best practices

- ELIXIR-SE
    - MIAPE for Proteomics, ISA for metabolomics. Any synergies?
        - MIAPE is a minimal requirements document from proteomics data, ISA is a means to report metadata, and is focused at a higher level (e.g., study design). These should therefore not necessarily be mutually exclusive.

- ELIXIR-FR: interface for biologists and clinicians: key interface to consider for proteomics; not just bring data to other integrate with other omics fields, but also to bring data to end-users outside of the core proteomics/mass spec community. Arguably, it is one of the key needs of the proteomics field right now, as its capabilities (and some of its limitations) are poorly understood by these neighbouring but non-(strictly-)overlapping communities right now!
- Several nodes are working on local data management systems for proteomics data (at least Belgium, France, and Sweden) which is a very good sign - arguably, this sort of system forms the foundation for many other components of a successful proteomics data management, analysis, and dissemination ecosystem.
- General discussion
    - Standardisation, data transfer, interoperability
    - Every node has their own system (LIMS, software).

        - Room for improvement.
        - ELIXIR has their own group dedicated for data transfer.
    - Experimental design related information is missing; also, interoperability with other omics.
        - Better metadata annotation is a need.
        - Limitations of resources in repositories/ curation is needed for further reprocessing.

Interactive session

In the interactive session, different aspects of identifying Stakeholders for the future of Proteomics, identifying their 'Needs and Challenges' and align them to ELIXIR platform and use-cases were discussed in groups or communally. The results are collectively filed [in one document](#). The following sections give a summary to the particular topics.

Identification of Stakeholders

A list of putative Stakeholders was individually compiled. All lists were communally consolidated:

1. Funding agencies
2. Regulatory bodies
3. Educators
4. Infrastructures
5. Publishers
6. Core Facilities
7. Bioinformaticians
8. Life Scientists
9. Industry
10. MS industry
11. Patients

Needs and Challenges

The needs and challenges were collected in Groups for each identified stakeholder. Needs are denoted with N, Challenges with E.

Group 1: Funding agencies, Regulatory bodies, Educators

- Funding agencies
    - Ensure data has an extended lifespan and lives longer than the funded project (FAIR data) (N)
    - Best practice recommendation for project evaluation and transparent/reproducible science (N)

    - Strategic planning for omics technologies and harmonized guidelines (N) across funding agencies (N)
    - Cost-efficient setup of omics core facilities (omics + bioinformatics) (C)
    - Recommendations for data management (C)
    - MS-based proteomics challenge is to convince FAs to proceed funding less matured technologies? (C)
- Regulatory bodies and ethics
    - Dealing with data privacy and security (C)
    - Audit/approval of software/pipelines in a clinical context? (C)
    - Harmonization of regulations across Europe (N)
    - System and workflow audit (N)
- Educators (train the researcher)
    - Extend curricula to include: Data science, FAIR data and reproducibility for comp. bio/bioinformatics curricula (N)
    - Synchronize curricula (e.g., in bioinformatics) (N)
    - Widely available teaching materials (N)
    - Uniformization of the lecture material, MOOCs, SPOCs and concepts (controlled vocabulary) (N)
    - Training the educators (N)
    - Teaching concepts rather than application (C)

Group 2: Infrastructures, Publishers, Core Facilities

- **Infrastructures**:
    - compatibility / interoperability of tools (N)
        - Missing file format conversion (C)
        - Missing standards? (C)
        - Standardized APIs, Web Services, message driven communication (C)
    - high-quality interoperable proteomics data (N)
        - Computer-readable meta-data including experimental conditions (C)
        - Missing minimal Quality standards (C)
    - let infrastructure combine their data with Proteomics data (N);
    - mapping large-scale experiments results to single protein entities / accessions (N)
        - efficient data structure for searches across whole infrastructure (C)
    - Use of accepted ontologies (C)
    - Secure, safe IT infrastructure compliant with data privacy (N)

- **Publishers**:
    - Quality Checks (N)
    - Reproducibility Checks (N)
    - Stable / Sustainably funded Data Repositories (N)
    - List of Rules / Guidelines about details for Proteomics data publication (N)
        - Harmonization of rules for data publication between journals (C)
    - Increasing need of storage (C)
    - Growing demand for open access => new business model? (C)

- **Core Facilities (similar to Infrastructures, but more fine grained)**:
    - Data Management System for protocol management, efficient raw data and results management (N)

        - Automated Workflows (N)
        - Automated Quality Control (N)
        - Compatibility of tools with formats from different instrument vendors (C)
        - Traceability of used algorithms (versions, parameters,...) & produced datasets (C)
        - Easy plug-in of new technologies into existing workflows (C)
    - Give analysis results in a very short time (N)
    - Cost-effective analysis (N)
        - Allow enough time for the analysis / quality needed (C)
    - Data / Result Visualization Tools (N)
    - Easy / simple visualization of overview / excerpt / important part of a data set (N)
    - Robust, sustained & user-friendly tools (N)
    - After version updates of tools / databases not too much results shall change (C)
    - Difference in interests about data publication (researchers want data to be secret long (C)
    - Many labs are developing own tools for component identification / annotation => should be aligned (N)
    - Benchmarking tools & datasets (intra- and inter-laboratory) to evaluate the quality of workflows (C)
    - Standardization of protocols and analyses between core facilities (C)

Group 3: Bioinformaticians, Life Scientists

- Bioinformaticians:
    - Quality control (measurements, annotation, support in PRIDE, etc.) at various levels (NC)
    - Automatic extraction of metadata from papers (NC)
    - Standardization of metadata (NC)
    - Quality control (measurements, annotation, support in PRIDE, etc.) at various levels (NC)
    - Access to computation and storage resources (N)
    - Quantitative proteomics comparability (C)
- Life scientists:
    - User-friendly interfaces (N)
    - Proteomics data in UniProt and other databases
    - Proteomics in model organism communities/resources (NC)
    - Local data management systems, LIMS and ELNs – capturing relevant information, guidelines for LIMS and ELNs (N)

Group4: Industry, MS industry, Patient

- Industry:
    - Reliability, robustness and reproducibility (C/N),
    - Quality assurance and control (N),
    - Lack of reference data for non biomedical industries [C],
    - Specific challenges of synthetic peptides [C],
    - High investment and short life span of instruments / technology and software [C],
    - High promises are not always met [C]
- MS Industry:
    - Maximizing sales (N),
    - Compatibility /interoperability [C] ,
    - Following the standards established by the community [N]

- Competition in terms of bioinformatics tools [C],
- Free academic tools competing with commercial tools [C]
- Conflict between their own commercial needs and the openness needs of the user community (N)
- Hardware / software compatibility [C]

- Patient (or rather the hospital / physician?):
  - Personal data / privacy [C],
  - Communication / visualisation of results (N),
  - Comprehensive integration of other omics layers and meta data (N)
  - Public awareness of proteomics opportunities [C]
  - Interpretability [C]
  - Robustness of mass spec for hospital, QC & QA [C]

Working across *omics

An open conversation was held over working across different *omics. A concluding summary on Metabolomics/Proteomics exchange can be found following.

Metabolomics and proteomics:

- The metabolomics community is working towards establishing a Use Case in ELIXIR. There are many similarities to our alignments with Training, Compute, Data, and Interoperability.
- TRAINING: As a result of a ELIXIR-UK funded workshop on Training in Metabolomics, the European Metabolomics Training Coordination Group was established: http://www.emtrag.eu/
- TOOLS: “PhenoMeNal” is a large € 7 million Virtual Research Environment project which has many general applicable components for the Proteomics community http://phenomenal-h2020.eu/home/. The idea of ELIXIR-DK is to organize a hackathon on integrating Metabolomics tools well in ELIXIR’s bio.tools.
- DATA: PhenoMeNal and the Go FAIR initiative will organize a workshop on Establishing a Metabolomics and Phenomics node in the European Open Science Cloud on March 9-10, more information is to be found in this form.
- INTEROPERABILITY: a consortium with members present are currently preparing a second stage H2020 Infrastructure Metabolomics Starting Community initiative (deadline March 29th 2017), integrating the leading facilities in Europe (comparable to PRIME-XS) for standardisation of wet analytical procedures, which includes Work Packages on FAIR data and Training.
- COMPUTE: is also a big issue in large-scale Metabolomics, the Imperial Phenome Centre is producing alone already several PB per year. There is ongoing discussions with EBI on how to accommodate the COMPUTE and STORAGE needed for this.
- Local metabolomics contact point for interoperation and outreach:
  - UK: EBI Metabolights (Claire o’Donovan), Birmingham (Mark Viant) and Imperial College London (Jake Pearce)

- Germany: University of Jena (Chris Steinbeck) and Halle (Steffen Neumann)
- France: CNRS, INRA and CEA
- Estonia: University of Tallin
- Greece: FORTH (Maria Klapa)
- Netherlands: ELIXIR-NL (Rob Hooft, TC and Jaap Heringa, Head of Node) and Leiden University (Thomas Hankemeier)
- Belgium: ELIXIR-BE (Frederik Coppens, deputy head of node and technical coordinator), VIB (Bart Ghesquiere, Head of Metabolomics Expertise Center). Follow-up done by Merlijn.
- Denmark: ELIXIR-DK, it would be nice to have a thematic hackathon to add metabolomics software to bio.tools (Veit Schwämmle). I can help with the organization

Day 2 Notes

Integration within Elixir

The second day started with recapturing the collected needs and challenges for the identified stakeholders, ordering them in a matrix layout and discussing the alignment of stakeholders against common needs and challenges.

Recommendations

Elixir recommendation needs:

- Tools in containers/dockers/whatever as basis for comparability
- Guidelines for parameter adaption and documentation
- Guidelines for tool development
    - Technical aspects of documentation, code cleanliness, unit testing
    - Checking for good practice development

- Gold- or at least common-standard datasets as basis for comparability

Collection of defined workflows (tools+parameters) for reproducibility
Horizontal benchmarks (or challenges/contests) using common guidelines

Repository/reddit board for tool ideas/interest

- Needs a mild form of moderation and connecting
- Indicating
- Connecting data producers to potential developers

More concrete tool category requirements:

- Data reduction in hires-MS
- Cross omics tools/metaproteomics
- Tools for integrating MS data in "other" proteomics (e.g. structural)
- Protein-quantification with protein-inference
- Quality control output for established tools

Priorities and discussion

Basing on the collected and aligned needs, challenges, stakeholders, and platform integrations, a discussion was held about the priorities of the individual topics and a structure of coverage for a white-paper on proposed future strategies laid out. Finally, a discussion was held on the integration with other disciplines and the engagement with external stakeholders (non-ELIXIR). Many points were taken up again, occurring frequently in earlier discussions. The needs and challenges were organised and clustered in wider areas. Following, a prioritising vote was taken.

Paper

- Strategic recommendations from funding
- Multi-omics
- Reproducibility
- Infrastructure
  - Cloud
  - Data repositories - data management
- Tools
  - Accessible
  - Sustainable
  - Discovery
- Educations

Ranking

The list of the top-ranked areas that could form the basis of a potential future proteomics use case in ELIXIR, sorted by the number of votes received.

1. The first cluster of activities corresponded to "Across-omics approaches" (11 votes), involving topics such as data integration of proteomics and other omics data (e.g. genomics and metabolomics), gene and protein expression correlation, and development of data standards for this across-omics interface. A closely related cluster

of activities was “Cancer proteomics” (receiving 4 additional votes), including topics such as support for clinical proteomics data (with large cohorts) and cancer multi-omics (proteogenomics) studies.

2. “Proteoforms and post-Translational Modifications (PTMs)” (11 votes). This cluster of activities included topics such as handling and validation of proteoforms, improvement of the linking between proteoforms, genes and metabolites, and activities devoted to discern unexplained spectral signals.
3. “Quality Control (QC) activities” (8 votes). This cluster focused in activities enabling automatic pipelines for QC of proteomics data at different levels.
4. “Workflow pipelines and activities related to cloud infrastructure” (7 votes). The concrete activities outlined here could be summarized as the development of robust, scalable, user-friendly, integrated, QC-controlled, data analysis pipelines/workflows, ideally enabling the use compatible cloud infrastructure (that could also be used for data storage).
5. “Protein quantification and statistics” (6 votes), including topics such as the improvement of protein inference, the use of shared peptides and enhanced data integration/harmonisation for quantitative proteomics approaches.
6. “Metadata/Standardisation/Annotation/Data management” activities (5 votes), including topics such as the improvement of annotation of proteomics datasets, in particular in data repositories, the development of Laboratory Information Management Systems (LIMS), standard data formats and development of best data management practises.

Wrap-up

- Spreadsheet to collect feedback
