# Supplementary material for: A community proposal to integrate proteomics activities in ELIXIR
Source: F1000Res. 2017 Jun 13;6:ELIXIR-875. [Version 1] doi: 10.12688/f1000research.11751.1 (PMC5499783; doi:10.12688/f1000research.11751.1)
Supplement: Peer review history

# Peer review: Referee response for version 1

**DOI:** 10.5256/f1000research.12693.r23920

**Published:** 2017-07-05

**Authors:** Chuming Chen

**Affiliations:** 1 Center for Bioinformatics and Computational Biology, University of Delaware, Newark, DE, USA

This white paper presents a framework for integrating computational proteomics into ELIXIR as the result of a strategy meeting on ‘The Future of Proteomics in ELIXIR’ that took place in March 2017 in Tübingen (Germany). The meeting discussions lead to six priority areas: Multi-omics approaches; Proteoforms and PTMs; Quality Control (QC) activities; Data analysis workflows and cloud computing; Protein quantification and statistics; Metadata, standardization, annotation, and data management. The priority areas are well aligned with the current ELIXIR platforms: Data platform; Tools platform; Interoperability platform; Compute platform; Training platform and can be integrated into ELIXIR. The paper is well structured and written.

 Minor change: 

 On page 7 of PDF version of paper, paragraph "2 - Tools platform ...":

 "it is worth highlighting that there are around 350 tools proteomics tools represented in this resource already", the first "tools" should be removed.

I have read this submission. I believe that I have an appropriate level of expertise to confirm that it is of an acceptable scientific standard.

# Peer review: Referee response for version 1

**DOI:** 10.5256/f1000research.12693.r23454

**Published:** 2017-06-28

**Authors:** Felix Elortza

**Affiliations:** 1 CIC bioGUNE, CIBERehd, ProteoRed-ISCIII, Derio, Spain

Proteomics has reached a point in which bioinformatics is of paramount importance for the process to extract the maximum information out of the enormous amount of data obtained in practically any study. The scientific community in general and proteomics in particular is eager to get new and effective solutions that may implement that way. In this white paper manuscript, Vizcaino *et al. *explain the ELIXIR initiative and the importance of integrating proteomics in this European Research Infrastructure. Authors cover in a detailed way what was covered on the meeting that took place in Tübingen (Germany) on February 2017 under the name “The future of proteomics in ELIXIR”. The paper is well written and structured. Conclusions are clearly stated.

I have read this submission. I believe that I have an appropriate level of expertise to confirm that it is of an acceptable scientific standard.
